# Co‐Design With Mothers and Professionals to Enhance Care for Mothers Experiencing Adversity: Development of the METIC Model

**DOI:** 10.1111/hex.70711

**Published:** 2026-06-01

**Authors:** Lauren Elizabeth Lines, Amelia G. Scott, Tiffany Conroy, Sarah Hunter

**Affiliations:** ^1^ Caring Futures Institute, College of Nursing and Health Sciences Flinders University Adelaide South Australia Australia; ^2^ Southern Adelaide Local Health Network Adelaide South Australia Australia; ^3^ Preventative Health SA Government of South Australia Adelaide South Australia Australia

**Keywords:** adverse childhood experiences, co‐design, psychosocial support systems, qualitative research, stakeholder participation

## Abstract

**Introduction:**

Co‐design is increasingly used to partner with key stakeholders to facilitate user‐driven improvements to health and social care services. Successful co‐design relies upon stakeholder partnerships that promote empowerment and value diverse perspectives—which can be challenging when stakeholders are from diverse or marginalised backgrounds. Within this context, our research aimed to co‐design a research and translation action plan to enhance non‐stigmatising care for mothers experiencing adversities during pregnancy or their children's early years.

**Methods:**

Theoretical frameworks (social constructionism, knowledge translation and experience based co‐design) and local guidelines (ethical and consumer participation) informed successful co‐design with stakeholder groups with power differentials and experiences of marginalisation. We developed the novel MEdiated Trauma‐Informed Co‐design (METIC) model remain sensitive to power differentials and experiences of trauma by embedding trauma‐informed approaches.

**Results:**

We engaged two stakeholder groups (professionals and service users) to promote equity and enable safe spaces for discussions using the METIC approach. The METIC approach includes five steps: (1) building relationships and triangulating evidence, (2) recruitment and data collection, (3) data analysis and feedback, (4) sharing outcomes and (5) ongoing engagement. Key learnings included the importance of taking time to build trusting relationships and using communication strategies tailored to diverse audiences, such as easy‐read visual summaries.

**Conclusions:**

Success was demonstrated through the co‐design of an accessible research and translation action plan, which received overwhelmingly positive participant feedback. Key methodological challenges for future consideration include how researchers should respond to broader and systemic factors that impact equitable engagement in co‐design.

**Patient or Public Contribution:**

Members of the public contributed to the conceptualisation of this research as described in detail within the manuscript.

**Trial Registration:** Not applicable.

## 1 Introduction

Co‐design has a variety of definitions and applications within health and social care research [1]. Broadly speaking, co‐design involves active involvement of key stakeholders in design and/or implementation of products, services and research by combining lived experiences and professional expertise [2], or put simply, co‐design is ‘designing with, not for, people’ [3]. The benefit of co‐design is to enable individuals to have input into research that impacts them to ensure stakeholders' priorities are considered, and subsequent solutions are better tailored to service users' needs [4, 5]. Co‐design methods are flexible and can be underpinned by different theories appropriate for a range of research areas, aims and contexts [2]. However, application of research methods without conceptual clarity can risk ‘watering down’ the core principles of co‐design [2]. There is often inadequate detail provided when reporting co‐design research, with Avila‐Garzon and Bacca‐Acosta [6] recommending that future researchers comprehensively report stakeholders, phases and activities to enable methodological replication.

While co‐design lacks a standard theoretical base or methodology, certain principles and values should underpin co‐design research to enhance the likelihood of successful outcomes [1, 4]. First, co‐design emphasises participant empowerment by partnering with stakeholders to develop ideas, knowledge and solutions for real‐world problems which directly impact them [2, 7]. In health and social care research, co‐design often involves working with people who have lived experience of a diagnosis, accessing a service or may use services in the future [8, 9]. One Australian framework published by the Agency for Clinical Innovation [5, p. 5] is used widely in healthcare and defines co‐design as bringing together stakeholders (parents, carers, families and health workers) to inform service improvements with an ‘equal and reciprocal relationship between all stakeholders, enabling them to design and deliver services in partnership’. Within this framework, the key principles of co‐design are (1) equal partnerships, (2) openness, (3) respect, (4) empathy and (5) designing together, which are underpinned by team capability for collaboration and empowerment [5].

Guided by the Australian Agency for Clinical Innovation's [5] and Experience Based Co‐Design [10] definitions and principles of co‐design, our project aimed to co‐design a research and translation action plan to enhance non‐stigmatising therapeutic care for mothers experiencing adversities during pregnancy or their children's early years (ages 0–5 years). Parents are responsible for providing safe and caring home environments that meet their children's health and developmental needs, yet psychosocial adversities such as mental health challenges, disability and socioeconomic disadvantage are beyond individual families' control and require outside support [11]. Parents with trauma histories are more likely to experience vulnerabilities that predispose them to lifelong adversities and/or further traumas during adulthood [12, 13]. Family adversities have lifelong, cumulative impacts for children and children living in families with multiple adversities are more likely to experience abuse or neglect [14]. Pregnancy and the transition to parenthood are times of increased support needs, but help‐seeking is stigmatised, and mothers are implicitly blamed by systems that emphasise individual responsibility without due consideration of broader contextual factors [13, 15].

## 2 Overview of the Issues

Blaming of mothers and parents is evidenced by statutory child protection systems which focus on individual ‘cases’ of maltreatment without considering the broader interplay of factors beyond parents' control that contribute to child maltreatment [14, 16]. When broader factors place children at risk of poor outcomes, mothers are framed as ‘perpetrators’ and children ‘victims’ by a system that itself victimises mothers by failing to provide adequate wrap‐around support [17, 18]. In this context of blame, marginalisation, inadequate support and ongoing cycles of adversity, mothers have little voice or power to influence outcomes, often feeling obliged to comply with professionals' demands to avoid negative labels or child protection intervention [19, 20]. Professionals are not always equipped to manage challenges such as collaborating across siloed services and balancing children's safety with women's support needs [21, 22]. Consequently, to equip professionals to more effectively support mothers and enable better outcomes for children, we need to understand service users and professionals' unique perspectives.

Despite emerging evidence of the need to meaningfully engage with stakeholders in health research, the authors were not aware of methodological guidelines for co‐designing with two stakeholder groups with significantly different power differentials. We recognised that power imbalances between service users, professionals and researchers could reduce service users' confidence to contribute or even lead to service users' perspectives being dismissed or devalued [23]. In contemporary Western contexts, professional knowledge is privileged as ‘objective’ and ‘scientific’, while lived experience is construed as less valid or ‘irrational’ [23, 24]. Professionals may not feel comfortable speaking candidly about their experiences in the presence of service users—possibly choosing to sanitise or censor their perspectives. For example, professionals may struggle to balance scientific accuracy with the need to be sensitive when discussing mental health challenges or substance use [25]. Many of our stakeholders were likely to have experienced trauma; around 70% of the general population have experienced a traumatic event, with higher prevalence in marginalised populations—such as service users who have experienced adversities [26]. Although there are existing Australian and international guidelines, none were bespoke for working with our stakeholder groups of service users (mothers) and professionals.

The perspectives of service users and professionals were crucial, but to avoid placing undue burden on either group, we provided choices to be involved in co‐design at different levels. The International Association for Public Participation describes five levels of public participation from simply **informing** people, to **consulting** to obtain feedback, **involving** people to ensure their views are understood, **collaborating** to partner in decision‐making or **empowering** people to enact change [27]. Therefore, to maximise the impact of stakeholder views while balancing the burden of time, we provided participants with choices to be consulted, involved and/or collaborate. Examples of **consulting** included participating in an initial interview, while **involving** meant participants were able to impact the research findings through their feedback on our findings. **Collaborating** continues with individuals who remain in touch via consumer networks or social media channels and/or choose to partner with us in ongoing work as participants or consumer researchers. We used a hybrid approach drawing upon a range of theories, principles and guidelines (refer to Table 1) to develop a bespoke co‐design methodology that aimed to maximise participants' choice and sense of safety to enable authentic involvement of all parties. The aim of this manuscript is to provide practical guidance from our own experiences to guide future co‐design with stakeholder groups where there may be power differentials.

**Table 1:** Key concepts, principles and guidelines informing the study and which step(s) they were applied.

Item	Context and/or definition/key elements	Steps where concept was applied
Social constructionism [28, 29]	Social constructionism recognises that knowledge is co‐constructed by the interactions between individuals and their sociocultural contexts, inclusive of human organisational structures. It critiques what is known to be true, recognising that there are multiple and divergent ways of knowing which become visible through how people converse about their experiences.	Step 1: Building relationships and triangulating evidence Step 2: Recruitment and data collection Step 3: Data analysis and feedback
Knowledge Translation (KT) Complexity Network Model [29]	The KT Complexity Network Model acknowledges complex interrelationships of individuals and systems through five principles essential to translating research evidence into practice. These non‐linear principles are (a) problem identification, (b) knowledge creation, (c) knowledge synthesis, (d) implementation and (e) evaluation.	Step 1: Building relationships and triangulating evidence Step 2: Recruitment and data collection Step 3: Data analysis and feedback Step 4: Sharing outcomes Step 5: Ongoing engagement
Trauma‐informed qualitative research [25, 30]	Guidelines for conducting trauma‐informed qualitative research for people who have experienced trauma, including preparing for the context, maintaining trust & safety and recognising potential impacts on participants and researchers. Trauma‐informed qualitative research is underpinned by five principles: (1) safety (physical and emotional), (2) trustworthiness, (3) choice, (4) collaboration and (5) empowerment.	Step 1: Building relationships and triangulating evidence Step 2: Recruitment and data collection Step 3: Data analysis and feedback Step 4: Sharing outcomes Step 5: Ongoing engagement
National Statement on Ethical Conduct in Human Research [31]	National guidelines that govern the ethical conduct of all human research undertaken in Australia.	Step 2: Recruitment and data collection Step 3: Data analysis and feedback Step 4: Sharing outcomes
Principles of co‐design [32]	Key elements of the co‐design process from the community sector perspective. Key elements include that co‐design is (1) inclusive, (2) respectful, (3) participative, (4) iterative and (5) outcomes focused.	Step 1: Building relationships and triangulating evidence Step 2: Recruitment and data collection Step 3: Data analysis and feedback Step 4: Sharing outcomes Step 5: Ongoing engagement
Spectrum of Public Participation [26]	International guidelines that define different levels of public participation in all forms of decision‐making which are inform, consult, involve, collaborate and empower.	Step 1: Building relationships and triangulating evidence Step 2: Recruitment and data collection Step 3: Data analysis and feedback Step 4: Sharing outcomes Step 5: Ongoing engagement
Experience Based Co‐Design (EBCD): A toolkit for Australia [9]	A toolkit that describes a methodology brings together service users' perspectives into co‐design processes that can improve healthcare quality, safety and users' experiences.	Step 1: Building relationships and triangulating evidence Step 2: Recruitment and data collection Step 3: Data analysis and feedback Step 4: Sharing outcomes Step 5: Ongoing engagement
Statement on consumer and community involvement in health and medical research [31]	National statement that aims to ‘guide research institutions, researchers, consumers and community members in the active involvement of consumers and community members in all aspects of health and medical research’ [31, p. 2].	Step 1: Building relationships and triangulating evidence Step 2: Recruitment and data collection Step 3: Data analysis and feedback Step 4: Sharing outcomes Step 5: Ongoing engagement
South Australia (SA) Health Sitting fees and reimbursement for external individuals [33]	Policy of the local public health service (SA Health) requiring reimbursement of stakeholders engaged for purposes of providing advice and guidance—inclusive of hourly rates. Our research team was external to SA Health so we were not obliged to follow these guidelines, however they are widely used within the local research community to inform reimbursement.	Step 2: Recruitment and data collection Step 3: Data analysis and feedback
Braun and Clarke's Reflexive Thematic Analysis [34]	Reflexive thematic analysis can be used across the qualitative paradigm summarise, provide rich description and interpretation of a data set. There are six key stages: (1) familiarisation with data set, (2) coding, (3) generating initial themes, (4) developing and reviewing themes, (5) refining, defining and naming themes and (6) writing up.	Step 3: Data analysis and feedback

## 3 Methods

### 3.1 Theoretical Frameworks

This research was underpinned by a social constructionist lens, which conceptualises knowledge as constructed and sustained by human interactions within organisational structures [28, 29]. Knowledge is visible through the language used to discuss human experiences, inclusive of social issues like child maltreatment or family violence [15]. Language used to describe child maltreatment manifests through interpersonal social interactions within dominant structures such as mainstream and social media, organisational procedures and government policies [30, 31]. These interpersonal interactions shape and are shaped by the complex interplay of individuals' day‐to‐day interactions embedded within organisational structures and sociocultural environments [28]. Within these interpersonal interactions, family adversities and child maltreatment are constructed in vastly different ways by parents and professionals.

In the context of child maltreatment and family violence, examples of dominant professional, societal and organisational discourses include ‘vulnerable’ children, ‘victims’ of violence and ‘perpetrators’ of abuse [19, 32]. Discourses are used by people in particular roles to construe the world in certain ways [33]; for example, criminal justice discourses emphasise protection of vulnerable child victims from perpetrators without due recognition of broader contextual factors [30, 34]. When socially constructed institutions prioritise individual parent characteristics over broader factors, it produces blame and stigmatisation, reduces help‐seeking and does not address root causes of abuse [35]. Professionals within socially constructed institutions make consequential decisions about parental capacity and child safety, potentially leading to child protection intervention.

Dominant discourses of individual parental responsibility are sustained by large organisations which have power within their socially constructed domain [33]. For example, the criminal justice system determines right from wrong, while medical professionals are ‘experts’ in health and illness [29]. These dominant discourses give greater power to mainstream ways of understanding the world, but children, parents and professionals draw upon alternative discourses to describe their experiences in preventing and responding to abuse. For example, children and young people describe themselves as active agents in resisting abuse [36, 37, 38]. Similarly, women oppose labels of being a ‘bad mother’ by emphasising how they love, care and provide for their children [19, 39]. Biomedical conceptualisations of women as primarily responsible for children's health and well‐being are unjust because they fail to include fathers, extended family and broader socioenvironmental factors shaping child health and women's agency [40, 41]. Professionals also construct children and families in particular ways shaped by their own values, beliefs and experiences [17, 42]. In recognition of the diverse ways that children, mothers and families are conceptualised, it was essential to understand professionals' lived experience to inform our research and translation action plan [43].

Embedded within our approach was a Knowledge Translation (KT) lens to acknowledge the ongoing challenges of translating research evidence into practice. KT refers to processes of creation and application of knowledge into dynamic, real‐world contexts by navigating the complexities of organisational cultures and structures that influence health professionals' practice [44, 45]. We were guided by the KT Complexity Network Model which involves five non‐linear principles of (a) problem identification, (b) knowledge creation, (c) knowledge translation, (d) implementation and (e) evaluation [46]. The KT Complexity Network Model aligns with social constructionism by acknowledging the complexity of interrelated policies, practices, cultures and discourses within health and social care services. Our project drew from diverse stakeholder perspectives to commence (a) problem identification, (b) knowledge creation and (c) knowledge synthesis which are foundational for future (d) implementation and (e) evaluation work.

In addition to the philosophical and theoretical basis, we integrated other principles as outlined in Table 1. Social constructionism and the KT Complexity Network Model provided our guiding theoretical lens for engaging with stakeholders. We also used the Spectrum of Public Participation and Experience Based Co‐Design to operationalise working with stakeholders [10, 27]. Finally, the other principles in Table 1 facilitated a trauma‐informed approach that was flexible to how our stakeholders wanted to collaborate and manage power differentials. Other researchers may have equivalent local guidelines, but we have summarised the basis for our research understanding that future researchers may select alternative local guidelines.

All concepts are applied within the context of the authors' positions of relative socioeconomic and institutional privilege, including financial security, compared with many service users. All researchers are female, working at a university and have backgrounds in nursing (L.E.L. and T.C.), psychology (S.H.) and dietetics (A.G.S.), which influenced co‐production and interpretation of data. We acknowledge these influences and critically reflected upon how our positionality shaped construction of knowledge and interpretation of professionals' and service users' perspectives.

### 3.2 Overview of the MEdiated Trauma‐Informed Co‐Design Model

**Figure 1:**
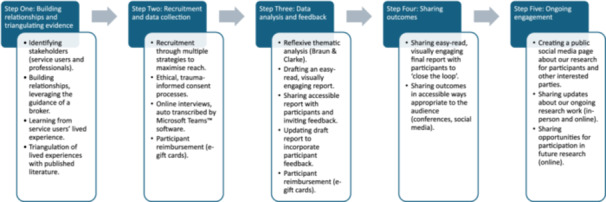
Outline of key steps and activities within METIC co‐design.

Through blending approaches, we successfully undertook a 5‐step process (summarised in Figure 1), called the MEdiated Trauma‐Informed Co‐design (METIC) to reflect its key elements of being (1) researcher‐mediated and (2) trauma‐informed. Its aim was to engage key stakeholders with significant power differentials and facilitate equal contributions to research outcomes. Navigating power differentials is a continuous and nuanced process; crucial elements are building and maintaining relationships underpinned by trauma‐informed principles and strategies to enable broad accessibility. Trauma‐informed principles inform engagement that promotes (1) safety (physical and emotional), (2) trustworthiness, (3) choice, (4) collaboration and (5) empowerment [26, 47]. Trauma‐informed principles require researchers to enact these principles throughout their interactions with participants, especially given the relational nature of qualitative research. Trauma‐informed principles are essential not only for participants with known trauma experiences but also for participants who choose not to disclose trauma. Trauma prevalence is very high; around 70% of the general population and even higher in minority populations or people who have been marginalised [26]. Next, we describe our results and key learnings that arose through application of the METIC model.

## 4 Results

### 4.1 Step 1: Building Relationships and Triangulating Evidence

Critical to this research were relationships built on authentic trust and rapport which leveraged long‐standing connections with stakeholders. One author (S.H.) had a joint position with a government department where colleagues had strong existing relationships with a local group of service users. Dr Hunter and a colleague (separate from the authorship team) facilitated access to service users through existing relationships when researchers otherwise might not be readily welcomed. Power imbalances within this group of service users were further mitigated through a co‐facilitation model whereby a service user co‐facilitates with a social worker through negotiated partnerships.

The service users were individuals who had lived experiences of accessing support for adversities that impacted their own and their families' health and well‐being. The group were employed by a government department to provide advice about how family support services can best enable children and families to live safe and well [48]. We met with this group on several occasions to discuss our research ideas and explore service users' perspectives. We informed service users prior to (via meeting agenda) and at the beginning of the meeting that our purpose was to gain feedback that would inform our future research. Prior to each meeting, we liaised with the co‐facilitator as a broker to explore whether our discussion points were appropriate, understandable and sensitive to service users. Social constructionism informed our discussions because we were seeking to understand service users' co‐constructed meanings rather than seeking objective facts.

Service user attendance in meetings was approximately 6–10 parents/caregivers, with some variability over time due to individual availability, and service users leaving or joining the group. During our first meeting with the group of service users (approximately 60 min, in‐person), our discussion points included how health and welfare professionals' language and interactions impacted how they felt about support services. Service users shared stories of when professionals' communication and interactions were impactful—positively and negatively. In our second meeting (approximately 30 min, online), we summarised discussion points from the first meeting and how this informed our research—specifically writing a discussion paper [49], and planning a research project. Service users' feedback was positive, so we proceeded with an international systematic review [20] to triangulate with knowledge arising from lived experience build a broader understanding parent/caregiver experiences.

It was not until our third meeting (in‐person, approximately 60 min), that we asked for formal participation in research activities (refer to *Recruitment*). Activities within this process represented consultation, involvement and collaboration [27] depending upon each individual's choices to make individual contributions.

### 4.2 Step 2: Recruitment and Data Collection

Next, we identified two key stakeholder groups, which were (1) mothers (service users) who accessed support for adversities that impacted parenting, and (2) health and welfare professionals who support these mothers. We chose mothers specifically because women are typically responsible for most childrearing and were the target population for our research funding (see acknowledgements). Mothers were eligible to participate if they had experienced an adversity during pregnancy and/or while their child was aged up to 5 years old in Australia. Adversities included broad factors that are known to negatively impact child health and development, including parental poor mental/physical health, substance use, housing/financial insecurity and family violence [50]. Professionals were eligible to participate if they had experience supporting mothers who met the eligibility criteria in Australia. Ethical approval for this research was granted by Flinders University Human Research Ethics Committee (project number 6535) and research was conducted in accordance with the National Health and Medical Research Council [51] ethical guidelines for human research.

#### 4.2.1 Recruitment

Given our existing connection with the group of service users, we advertised our research project through this group with permission, and subsequently through other national parent groups and via social media (Facebook). We originally intended to recruit 10 service users and 10 professionals based on likelihood of achieving saturation [52], but two professionals (colleagues) wished to be interviewed together—bringing the total number of professionals to 11. The number of participants was established in advance on the basis of data saturation occurring within 12 interviews [53], and the majority of new codes arising within the first 20 interviews [54]. Recruitment of professional participants included through professional networks, such as seminars, newsletters, social media and snowball sampling. The most successful strategy to recruit professionals was via snowball sampling where participants recommended the study to colleagues.

#### 4.2.2 Sample Characteristics

Recruitment of participants can be challenging when research involves people who have experiences of trauma, socioeconomic adversities and/or engagement in child protection services [55, 56]. Although we experienced slow recruitment with service users initially, we reached our recruitment goal of 10 service users by expanding recruitment beyond the original group of service users through a maternal consumer advocacy group and social media (Facebook) support groups for mothers. Characteristics of participants recruited through different sources were noticeably different. For example, we observed that participants from the original group of service users typically had lower educational attainment and increased interactions with child protection services. Service users recruited via other methods in the expanded recruitment typically were not involved with child protection services and reported higher educational attainment and social capital. A broad overview of participant characteristics is provided in Table 2.

**Table 2:** Overview of participant characteristics.

Characteristics	Mothers *n* = 10	Professionals *n* = 11
Age (years)		
Under 35	*n* = 5 (50%)	*n* = 2 (18.18%)
35 or over	*n* = 5 (50%)	*n* = 9 (81.82%)
Educational attainment		
High school or lower	*n* = 3 (30%)	*n* = 0 (0%)
Vocational education	*n* = 3 (30%)	*n* = 1 (18.18%)
Bachelor's degree or higher	*n* = 4 (40%)	*n* = 10 (90.91%)
Employment status		
Not working	*n* = 4 (40%)	*n* = 0
Employed part‐time	*n* = 6 (60%)	*n* = 6 (54.55%)
Employed full‐time	*n* = 0	*n* = 5 (45.45%)
Number of children		
1	*n* = 4 (40%)	
2	*n* = 5 (50%)	
3 or more	*n* = 1 (10%)	
Professional discipline		
Healthcare		*n* = 9 (90.91%)
Social care		*n* = 2 (18.18%)

#### 4.2.3 Ethical Considerations

One challenge to recruitment was several service users who, without explanation, did not attend their online interview. These service users had telephoned the researcher soon after receiving project information from the co‐facilitator of the local group of service users and anticipated being interviewed immediately. However, in accordance with ethical guidelines, the researcher requested that service users review study information for informed consent and scheduled interviews for mutually agreeable later dates. Despite service users' initial enthusiasm, five service users did not attend the scheduled online interview or respond to follow‐up emails. Non‐attendance at scheduled interviews could be due to a lack of immediacy and subsequent lost interest. It is possible that some service users felt social pressure to contact the researcher after receiving information about the project from the co‐facilitator. In the context of people who have experienced trauma, they may have conscious or unconscious tendencies to conform with expectations of authority figures due to survival strategies built by traumatic circumstances [26]. Our consent procedures informed by national ethical standards [51] may have empowered some service users to decline in safe and trauma‐responsive ways.

#### 4.2.4 Data Collection

After consent, service users and professionals individually participated in a semi‐structured interview with the lead researcher (L.E.L.), which was audio recorded and transcribed via Microsoft (MS) Teams. As per the principles of co‐design, the researcher embodied core principles of respect and participation throughout the interview process [57]. Being respectful meant participants' perspectives were valued equally, while being participative meant building shared meanings through empathetic conversations [57]. The researcher built rapport with participants by enabling them to tell their story in their own words. Participants were invited to discuss only topics that felt comfortable and were empowered to tell their stories with minimal interruptions [26]. As such, participants led discussions with interview questions woven into conversations or natural pauses. Strength of rapport was demonstrated through participants sharing their vulnerabilities and challenges as a service user or professional. Participants were emailed copies of their transcripts after de‐identification and cross‐checking with the audio recording; none requested changes.

Trauma‐informed principles balanced research benefits with risks of vicarious trauma for researchers and re‐traumatisation of participants. To reduce participant harm, we intentionally focused questions for service users on experiences of accessing services rather than asking them to recount traumatic events (see supplementary online). For professionals, interview questions focused on their professional roles in supporting mothers, including their perceived strengths and challenges rather than recounting extreme or distressing cases (Supplementary Online). We recruited and interviewed service users who had previous experiences accessing support for adversities so they had time to process past traumatic events [48]. No participants became distressed, but all spontaneously discussed the positives of sharing their story—such as making a difference for future families. The two researchers (L.E.L. and A.G.S.) closely involved in data collection, transcription checking and/or analysis regularly met to reflect on each other's well‐being [58]. Neither researcher experienced distressing reactions but were able to reflect upon the resilience of participants and hoped that findings would improve future practice. The ability of researchers and participants to experience the research process as positive is reflective of our focus on resilience, hope and personal growth despite traumas [59].

### 4.3 Step 3: Data Analysis and Feedback

Data analysis was undertaken by two authors (L.E.L. and A.G.S.) informed by Braun and Clarke's principles of reflexive thematic analysis [60]. Transcripts were imported into NVivo (Version 12) and analysed inductively by L.E.L. and A.G.S., who met regularly to discuss and refine the codes and later, the draft themes. Draft themes were refined through discussion with all authors to establish agreement on coding processes and final themes, which were six key actions for future research and translation [43]. Within NVivo, we named each transcript as ‘mother’ or ‘professional’ to facilitate the interpretation of participants' social context. Mothers' and professionals' responses were all analysed inductively using the same coding structures to establish equal weighting of participants' views irrespective of their role as mother or professional.

Draft findings were presented in two formats to promote accessibility and minimise time burden. These formats were (1) an infographic to visually summarise key elements and (2) an easy‐to‐read, seven‐page document with images and contextual information. The infographic and document were emailed to participants with a request for them to provide feedback or discuss findings via email, comments/tracked changes, telephone or online (MS Teams).

**Figure 2:**
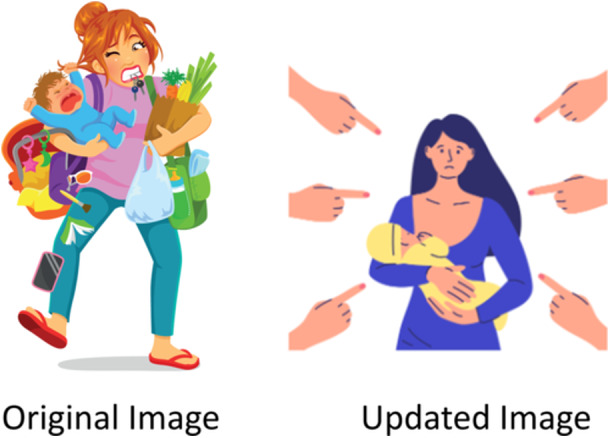
Example of image substitution based on participant feedback.

We anticipated that participants might wish to discuss findings in detail and recommend significant amendments. However, feedback was overwhelmingly positive, and participants felt their key messages were accurately portrayed [43]. From the initial 21 participants, 13 provided feedback and 8 did not respond. From these 13 participants, 11 (5 service users, 6 professionals) gave feedback by email, and two service users provided feedback through discussion via MS Teams. Out of the 13 providing feedback, seven recommendations were minor amendments to wording (*n* = 6) and substitution of images (*n* = 1). Although feedback was minor, it helped ensure the final report resonated with both professionals and service users—for example, an image of a harried mother was substituted with one depicting a neatly dressed mother (Figure 2). The predominantly positive feedback from participants demonstrated that our participative and respectful listening during Step 2 effectively interpreted participants' views.

### 4.4 Step 4: Sharing Outcomes

Finally, we emailed the amended easy‐to‐read, visual summary to participants, providing closure and a tangible outcome (Adobe PDF attachment). Within the final email, we again thanked participants and welcomed further suggestions to inform our future work. The final report was comprised of six stakeholder‐driven actions to inform our research team's ongoing work [43], and it was presented in a visual, easy‐to‐read format for accessibility.

Dissemination of research findings traditionally occurs within academic/professional communities, but not necessarily with service users. Out of respect for the valuable contributions of our service user participants, we complemented professional and academic dissemination with strategies more accessible to service users. Sharing outcomes with service users included invited presentations and discussions of our findings with the local service user network from Step 1 to show tangible project outcomes. We also shared findings via email and social media with other relevant service user networks; some invited and some researcher driven. Our easy‐to‐read, visual report is freely available online with a Digital Object Identifier (DOI) to enable ongoing public access to a permanent copy [43]. Although Step 4 represented the project endpoint, our engagement and relationships with stakeholders are ongoing.

### 4.5 Step 5: Ongoing Engagement

Some co‐design projects have a final endpoint for engagement where a product or outcome has successfully been developed. Examples of a finished product may include a health service, program or clinical guideline [9, 61]. However, achieving sustainable change within health and social care requires continuous evaluation through ongoing partnerships with service users [5]. Similarly, in our study, the final product was a research action and translation plan, which was not an endpoint but a starting point for ongoing work [46].

Throughout this project, both service users and professionals articulated strong personal and/or professional motivations to continue advocating for better support for service users and families. In recognition of participants' commitment to advocacy and the need for sustained ‘commitment to create change’ [57], we established opportunities for ongoing engagement for project participants and stakeholders more broadly. Ongoing engagement includes our relationship with the service users from Step 1 where we have an open invitation to provide updates, seek feedback and discuss future opportunities. In addition, developed a Facebook page to more widely share project findings and updates. This Facebook page creates an accessible online space for other stakeholders to learn about our work and consider partnering with us in ongoing projects.

#### 4.5.1 Key Learnings

Key learnings from the METIC model highlighted several interrelated considerations for co‐design with stakeholder groups experiencing power differentials. First, establishing and maintaining trusting relationships were foundational throughout and required significant time investment—particularly for participants with experiences of marginalisation. Second, researcher‐mediated engagement supported navigation of power differentials by creating safer spaces for authentic expression. Third, trauma‐informed principles required continuous enactment across all stages, especially by promoting safety, choice and accessibility. Fourth, flexible approaches to communication and engagement facilitated inclusivity and meaningful involvement of diverse participants. Finally, several challenges such as structural inequities inclusive of differential reimbursement highlighted the impact of systemic factors on equitable participation. Collectively, these learnings demonstrate that effective engagement with diverse stakeholders requires ongoing relational and flexible approaches beyond procedural application of methods.

## 5 Discussion

The aim of this manuscript was to draw upon the authors' experiences of meaningfully engaging two stakeholder groups in co‐design to provide practical guidance for future research. We developed a bespoke co‐design methodology aiming to attend to power differentials and maximise participants' sense of safety through a researcher‐mediated approach (METIC). METIC is founded upon principles of Knowledge Translation, social constructionism and trauma‐informed approaches, which are relevant to a variety of research contexts, including applied research, participatory action research, user‐centred design and community‐based participatory research. Although our project successfully achieved its aim of producing a co‐designed research action plan, we experienced several challenges that need to be considered by future researchers.

One element of trauma‐informed research is creating a space for people to share their experiences and the unique meanings of their experiences [27]. However, despite the authors' emphasis on curiosity and respect for participants' agency [62], we discovered that MS Teams automatically censored curse words (e.g., fu** and sh**). Upon reflection, we decided technological sanitising of participants' stories based on values embedded into software did not respect participants' narrative agency. The words people choose to tell their stories are important, and as qualitative researchers, it was not our role to censor participants' views.

We reimbursed participants to promote empowerment, equal partnerships and demonstrate respect for their expertise [5, 57]. As such, we budgeted for reimbursement following each element they contributed to, which were (1) initial interview, (2) feedback on draft and (3) feedback on final report. In accordance with local guidelines [63], hourly reimbursement rates were $35 AUD for service users and $50 AUD for professionals (based on the average hourly rate). Although we reimbursed all participants, this did not necessarily contribute to the equal relationships how we intended. First, local guidelines recommended different rates for service users and professionals [63]. Professional rates were informed by industry standard payrates but the rationale for service user rates was not stated.

One way that differences in reimbursement rates for professionals and service users could be interpreted is that healthcare organisations value professional knowledge more highly than knowledge from lived experiences, especially lived experiences from marginalised groups [64, 65]. In our study, the divide between service users and professionals was not completely linear because some service users were also qualified professionals. We offered all participants reimbursement in accordance with local guidelines and respected participants' autonomy regarding their choice of interview timing. Some professionals participated during paid working hours, while many service users participated during non‐working hours. As researchers, it was not our role to determine whether people participated during their working or non‐working hours, meaning some participants may have received both project reimbursement and their salary. Reflected within these circumstances were epistemic injustices, which are ‘forms of injustice that occur in the context of knowledge sharing and production within systems of power and oppression’ [66]. Epistemic injustice in healthcare contributes to the oppression of ‘patients’ through medical discourses that shape individual patient‐professional encounters informed by procedures and policies [64].

Qualitative interviews are commonly conducted via voice or video calls to enable greater flexibility and engagement with people who are geographically distant [67]. Both voice‐only and video calls can provide high‐quality data—including for sensitive topics [68, 69]. Our research aimed to use online video calls, but flexibility was important when participants had difficulty engaging with MS Teams. One service user had not previously used MS Teams but was able to download the application prior to the interview. In anticipation of possible technological challenges, the researcher prepared alternative means of interviewing and audio recording (mobile phone on speakerphone with a separate digital audio recorder) but fortunately, the participant was able to successfully use MS Teams. Another professional originally intended to use MS Teams but had an unexpected computer problem, which prevented access to MS Teams. The researcher did not have immediate access to the digital audio recorder but instead was able to record the conversation on speakerphone using the MS Word ‘dictate’ function to auto‐transcribe. These technological challenges demonstrate the importance of being flexible in the application of technology and responsive to accessibility needs.

During analysis, we observed many examples of participants openly sharing experiences of vulnerability. Service users described personally impactful incidents of adversities or accepted responsibility for actions impacting their children's health and safety. One service user without a reliable income described her resourcefulness in obtaining unwanted baby equipment left on the roadside by ‘rich people’—even though the interviewer (L.E.L.) with a steady income might also be construed as a ‘rich person’. Professional participants similarly shared experiences of vulnerabilities, including personal mental health challenges or balancing their empathy for clients within professional boundaries. In qualitative research, there is no single ‘truth’ but a multiplicity of truths where reality is co‐constructed within the intersubjectivity of research interviews [29, 70]. Participants' co‐construction of vulnerability demonstrates the strength of rapport with the researcher achieved through application of empathetic, trauma‐informed approaches.

Power imbalances are inherent within qualitative research, especially when aiming to understand the lived experiences of people who may be marginalised or impacted by trauma [26]. However, power relations in qualitative research are complex and must be critically acknowledged to understand their influence on the co‐production of data [71]. We responded to potential power differentials brought by co‐author Hunter, who through their government role, was able to facilitate access to service users by ensuring co‐author Hunter was not responsible for data collection or given access to identifiable information. Instead, the first author (registered nurse and early career researcher) collected data, and the second author, a dietitian and research student, de‐identified data and cross‐checked the transcripts. In doing so, we created space between authors who facilitated access to service users and data collection.

We similarly reflected upon and responded to potential power imbalances between professional stakeholders and the authors. As former clinicians (T.C., L.E.L. and A.G.S.), academics (all authors), one of whom is an internationally recognised professor (T.C.), we benefitted from our professional networks, reputations and credibility when asking professional organisations or services to share study information. However, to mitigate perceptions of power imbalances when collecting data from professionals and enable co‐construction on a more equal basis as a peer, Dr Lines (early career researcher and registered nurse), who had experience working with children and families, collected data, with co‐author Scott again de‐identifying the data. Previous research demonstrates the benefits of an interviewer who is a professional peer to promote rapport through shared knowledge, such as healthcare structures [72]. As such, by critically reflecting upon authors' roles within socially constructed hierarchies [28], we were able to mitigate power imbalances with both service users and professional stakeholders.

### 5.1 Strengths and Limitations

Strengths of this research included the commitment of all participating service users and professionals to advocate for better systems and support for mothers experiencing adversity. We captured a diverse range of perspectives including across professional disciplines and demographics. However, there are some limitations, which included that professional participants were more likely to represent views of those actively committed to change and working in strengths‐based ways. For example, participating professionals were more likely to represent a cohort with high capabilities for engaging therapeutically with mothers rather than professionals with lower capabilities, which may be experienced as coercive or disrespectful. Service users who had experienced negative or traumatic experiences with professionals and services may have been less open to participating in research. Another limitation was the lack of representation from mothers who were currently accessing supports for adversities. This population group might have additional insights, but will require further ethical considerations to enable safe participation.

## 6 Conclusions

In summary, co‐design research does not have an international consensus around its theoretical and methodological basis, meaning co‐design methods are variable and may not be replicable. Co‐design is increasingly applied to engage with diverse stakeholders, it is not always robustly reported to enable ongoing critique and methodological replication. In some co‐design projects, stakeholder groups may have significant power differentials, which can make direct interactions between groups difficult or inappropriate. We developed the METIC model as a novel way to engage with two stakeholder groups experiencing different power differentials, assisting all to have equal input. These five steps were (1) building relationships and triangulating evidence, (2) recruitment and data collection, (3) data analysis and feedback, (4) sharing outcomes and (5) ongoing engagement. Overall, our methodology was successful as research aims were achieved through the production of an accessible research and translation action plan, which received overwhelmingly positive participant feedback. Key methodological challenges for future consideration include researcher responses to broader systemic impacts on equity and stakeholder engagement in co‐design.

## Author Contributions

**Lauren Elizabeth Lines:** conceptualization, project administration, investigation, funding acquisition, writing – review and editing, writing – original draft, formal analysis. **Amelia G. Scott:** data curation, formal analysis, writing – review and editing, visualization. **Tiffany Conroy:** supervision, writing – review and editing. **Sarah Hunter:** conceptualization, supervision, writing – review and editing.

## Ethics Statement

Ethical approval for this research was granted by the Flinders University Human Research Ethics Committee (no. 6535) on 19‐10‐2023.

## Consent

All participants gave written informed consent prior to enrolment in the study.

## Conflicts of Interest

The authors declare no conflicts of interest.

## Permission to Reproduce Material From Other Sources

Not applicable.

## Supporting information

Supporting File

## Data Availability

Data not available—participant consent. The participants of this study did not give written consent for their data to be shared publicly, so due to the sensitive nature of the research, supporting data are not available.
