# Supplementary material for: Co‐Design With Mothers and Professionals to Enhance Care for Mothers Experiencing Adversity: Development of the METIC Model
Source: Health Expect. 2026 Jun 1;29(3):e70711. doi: 10.1111/hex.70711 (PMC13238825; doi:10.1111/hex.70711)
Supplement: Supplementary file 1 — Supporting File [file HEX-29-e70711-s001.docx]

Supplementary Online Sample Interview Questions

**Mothers**

1. Can you tell me about your interest in this research?
2. Can you tell me a little about yourself?
   1. Prompts: Educational attainment, employment, interests/activities.
3. Can you tell me a bit about your family?
   1. Prompts: number of children, their ages, their interests/activities, any major developmental/health diagnoses, social/family networks.
4. What challenges did you face that impacted your parenting during pregnancy and/or your children’s early years?
5. What health and social care services did you access? What professionals did you speak with (e.g. nurse, social worker, doctor)?
6. Which services were MOST helpful? Can you describe why?
   1. Can you give examples?
7. Which services were LEAST helpful? Can you describe why?
   1. Can you give examples?
8. What worked well about the support you received?
9. What did NOT work well about the support you received?
10. Did professionals’ communication or words influence your experiences? Can you give examples?
11. What changes would you like to see?
    1. Prompts: What could professionals do differently?
12. As you know, we are developing a report that will inform future research and practice change. If you could include anything at all, what would you put in there?
13. Do you have anything else you would like to share?

**Professionals**

1. Can you tell me about your interest in this research?
2. Can you tell me a little about yourself as a professional?
   1. Prompts: profession, qualifications, education/training, experiences, years of experience.
3. Can you tell me about your professional roles in supporting mothers experiencing adversity?
   1. Prompts: context of practice, types of adversities, current/previous roles.
4. Can you describe some examples of when you’ve supported mothers who are seeking support for adversities impacting their parenting during pregnancy and/or children’s early years?
   1. What is working well? What could be done differently?
5. Can you describe some challenges you’ve faced when supporting mothers who are seeking support for adversities impacting parenting during pregnancy and/or children’s early years?
   1. What could be done to better support you and other professionals?
6. How successful do you think you and/or your service is at supporting mothers who are seeking support for adversities impacting their parenting during pregnancy and/or their children’s early years?
   1. Prompts: what are the barriers/facilitators? What would you change?
7. What is most rewarding about your work in supporting mothers? What are the most challenging aspects?
8. What are your professional STRENGTHS when supporting mothers? What are areas you would like further development or service change?
9. Are there particular strategies, words or language you use to communicate with mothers, such as to reduce stigma or blame? Can you give examples?
10. As you know, we are developing a report that will inform future research and practice change. If you could include anything at all, what would you put in there?
11. Do you have anything else you would like to share?
